# A recombinant lysogenic bacteriophage inhibits *Salmonella* virulence

**DOI:** 10.1128/aac.01341-25

**Published:** 2025-12-23

**Authors:** Erick Maosa Bosire, Mudasir Ali Rather, Katherine E. Bell, Paulina D. Pavinski Bitar, Ranju Ravindran Santhakumari Manoj, Craig Altier

**Affiliations:** 1 Department of Population Medicine and Diagnostic Sciences, Cornell University 5922, Ithaca, New York, USA; Entasis, Big Bay, Michigan, USA

**Keywords:** *Salmonella*, invasion, diffusible signal factor, *cis*-2-hexadecenoic acid, *rpfF*, virulence

## Abstract

The gut environment includes an abundance of chemicals emanating from the host and the microbiota. To colonize animals, *Salmonella* uses gut chemicals as locational cues to ensure expression of energy-intensive virulence factors only when their production is necessary. *cis*-2-hexadecenoic acid (c2HDA), a member of the diffusible signal factor family of quorum-sensing signals, potently represses virulence-gene expression by *Salmonella*. Here, we report the construction and use of a recombinant bacteriophage that can establish lysogeny within *Salmonella* and induce it to produce c2HDA, thus repressing functions essential to its own virulence. We engineered the temperate phage P22 to favor lysogeny through transposon mutagenesis of the *sieB-esc* region and caused it to produce c2HDA by introduction of *rpfF* of *Cronobacter turicensis*, encoding the dehydratase/thioesterase required for c2HDA synthesis. We found that *Salmonella* harboring the lysogenic phage carrying *rpfF* produced c2HDA and repressed invasion-gene expression both endogenously and exogenously through secretion into the surrounding medium. Consequently, this phage reduced *Salmonella* invasion of epithelial cells by over 100-fold. We further found that both wild-type and c2HDA-producing phage administered orally to mice reduced *Salmonella* colonization of the gut, but that the phage carrying *rpfF* reduced gut inflammation more than did the phage without this gene. Collectively, our data show that a recombinant phage can be used as the vehicle for cytoplasmic delivery of c2HDA, thus providing a targeted means to manipulate *Salmonella* colonization of the gut.

## INTRODUCTION

As *Salmonella* traverses the animal intestinal tract, it encounters changing chemical environments created by both the host and the resident microbiota. This pathogen has evolved, however, not only to survive within these environments but also to recognize the changes and to use them as cues to promote its own survival and replication. In the proximal small intestine, *Salmonella* first encounters high concentrations of bile, which represses its type III secretion apparatus required for the invasion of intestinal epithelial cells (1, 2). Within the distal small intestine, bile dissipates and the fatty acid formate predominates (3), inducing this same apparatus and creating a nidus of cell invasion and ensuing inflammation (4, 5). In more distal parts of the intestine, chemical signals repressive to invasion again predominate, including short- and long-chain fatty acids (6, 7), allowing *Salmonella* to dedicate its energy instead to growth and replication (8).

Among the most potent chemical repressors of *Salmonella* invasion is *cis*-2-hexadecenoic acid (c2HDA) (9). This molecule is a member of the diffusible signal factor (DSF) class of quorum-sensing signals that is produced and recognized by several bacterial species (10). Among these species, the activity of DSFs is typical of quorum-sensing systems, regulating aggregation, motility, and the formation and dispersion of biofilms (11–14). Their effects on *Salmonella*, however, are quite different. At micromolar concentrations, c2HDA inhibits *Salmonella* invasion by binding and preventing the activity of three AraC-type transcriptional activators, HilD, HilC, and RtsA (9, 15, 16), that function collaboratively to induce invasion (17). The inactivation of these regulators thus profoundly reduces the expression of the genes needed for invasion and the penetration of epithelial cells (9, 15, 16).

The hallmark structure of DSF quorum-sensing signals essential to their function is an unsaturation at the second position in the *cis* orientation (10). Their production is catalyzed from existing pools of long-chain fatty acids by a dehydratase/thioesterase typically termed RpfF, which both creates the double bond and cleaves the thioester linkage of the fatty acid to acyl carrier protein (18). We have previously shown that *rpfF*, when expressed in a probiotic strain of *Escherichia coli*, produces c2HDA in sufficient quantities to inhibit the invasion of *Salmonella* when grown in co-culture. Furthermore, this c2HDA-producing strain can colonize the gut of chickens and reduce colonization of *Salmonella* in experimental infection trials (19).

These findings thus suggest that c2HDA might be used as a practical means to reduce *Salmonella* carriage in animals. The efficacy of this approach, however, likely depends upon the achievable concentration of this molecule at its site of action, the animal intestine. The question then becomes one of efficient *in situ* delivery. Here, we test a novel approach, engineering a recombinant lysogenic bacteriophage that harbors *rpfF* and produces c2HDA. We find that this phage induces *Salmonella* to endogenously produce its own c2HDA, thus efficiently repressing its own invasion. The repression of this essential virulence function results in attenuated invasion of epithelial cells and consequently reduced gut inflammation.

## RESULTS

### A recombinant lysogenizing phage produces a signal repressive to *Salmonella* virulence

**Fig 1:**
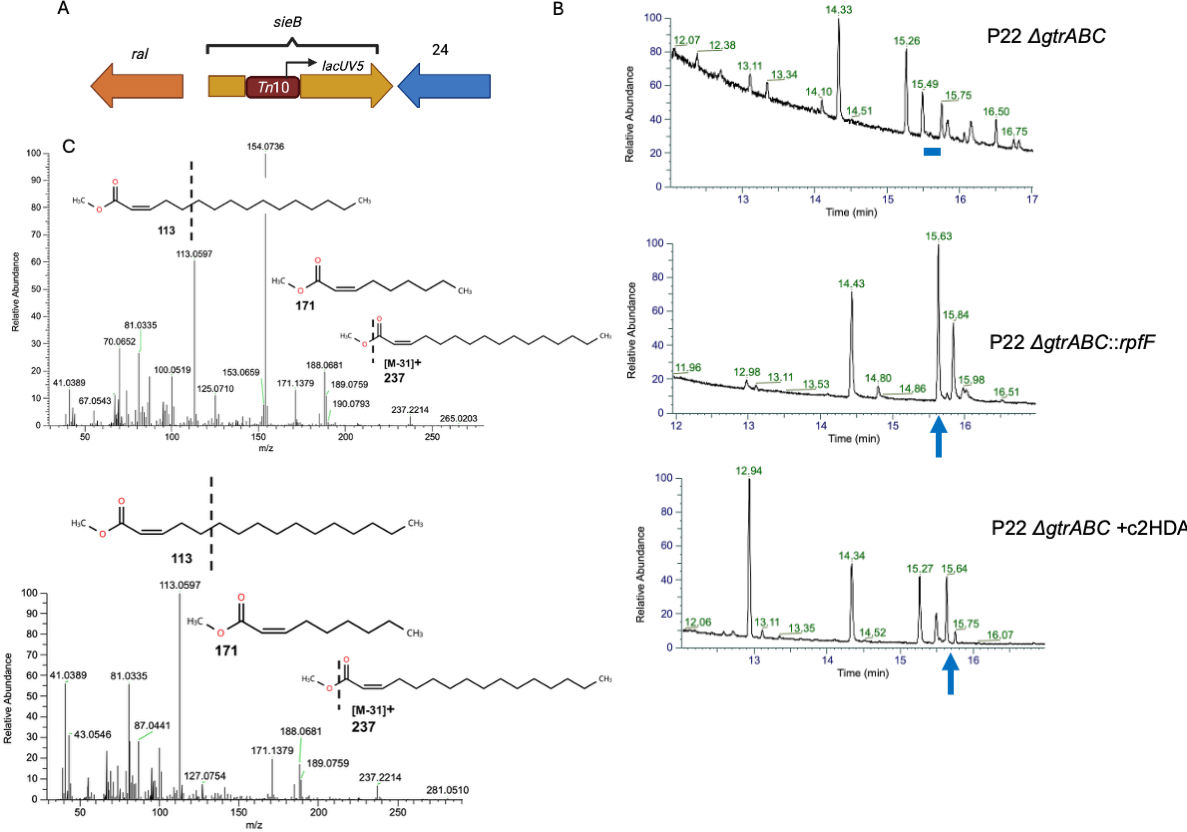
P22 phage lysogens carrying the *rpfF* gene produce c2HDA signal. (**A**) Construction of lysogens. Mutants with the transposon inserted at the *sieB-esc* region were more lysogenic. (**B**) GC-MS retention time analysis. The blue arrow shows the peak with the expected retention time for c2HDA (15.63 min, which represents the time this compound eluted from the chromatography column after injection) produced by the strain carrying P22 Δ*gtrABC::rpfF* (second from top), and a dash indicates the missing c2HDA peak in P22 Δ*gtrABC* strain (top). This retention time was confirmed by spiking c2HDA into extracts of P22 Δ*gtrABC* lacking the 15.63 min peak as indicated (bottom chromatogram). (**C**) MS fragmentation of the 15.63 peak for the commercial c2HDA (top) and that produced by the P22 Δ*gtrABC::rpfF* lysogens (bottom).

c2HDA, a member of the DSF class of quorum-sensing molecules, can potently repress *Salmonella* genes required for virulence (9). This molecule can be produced recombinantly in *E. coli*, effectively inhibiting the virulence of *Salmonella* in co-culture (19). We reasoned that this repressive system could be made more efficient by inducing *Salmonella* to produce its own c2HDA through infection by a lysogenizing recombinant phage engineered to generate the signal. We first created a mutant of the temperate phage P22, which infects *S*. Typhimurium and is widely used as a genetic tool (20), to favor lysogeny over lytic growth. Using random transposon mutagenesis, we selected for lysogens under conditions that typically promote lysis. One such mutant was found to harbor its transposon insertion in the *sieB-esc* region, 254 bp downstream from the *sieB* initiating codon. *sieB* encodes a superinfection exclusion system that prevents the lytic process by superinfecting phages (21). *esc* encodes a peptide within the *sieB* ORF that prevents the production of full-length SieB and thus protects the phage from its own destruction (22). An insertion in this region would thus disrupt both ORFs, allowing superinfection by infecting bacteriophage. To test whether this mutation induced lysogeny over lytic growth, we compared it to a derivative of P22, harboring a precise deletion of *sieB*, previously shown to produce lysogens at a frequency unchanged from that of wild-type P22 (23). Comparing transductants by selection on media containing kanamycin (as both phage are resistant to this antibiotic) to plaque-forming units (pfu), we found that our mutant phage yielded lysogens at a rate of 2.5 transductants/pfu, compared to 1.25 transductants/pfu for the control phage, yielding a twofold advantage. The mechanism by which this mutation favors lysogeny over lytic growth is unclear. It should be noted, however, that the insertion lies adjacent to gene *24* (24), a λ gene *N* homolog encoding an antiterminator required for the expression of lytic phage proteins (25) (Fig. 1A). The transposon itself carries an outward-facing *lacUV5* promoter (26), oriented in an antisense direction to gene *24*. Effects on gene *24* might thus reduce the efficiency of phage lysis. We next introduced into this phage *rpfF* of *Cronobacter turicensis*, encoding a dehydratase/thioesterase that places the distinctive *cis*-2 unsaturation characteristic of DSF quorum-sensing signals into saturated long-chain fatty acids (12, 18). The gene was expressed under the control of a constitutive promoter and replaced the P22 *gtrABC* operon, which causes *Salmonella* seroconversion (24), but is not essential for phage infection or replication. We tested this strain for DSF production, comparing it to an isogenic lysogenic strain harboring a P22 Δ*gtrABC* mutant without *rpfF*. GC-MS analysis showed that a long-chain fatty acid was produced by lysogens harboring the *rpfF*-encoding phage, but not by those with the control phage (Fig. 1B). Mass fragmentation pattern analysis further revealed this product to be c2HDA, based upon its comparison to the fragmentation pattern of the commercially available c2HDA (Fig. 1C). These results thus show that *Salmonella* can be infected with this recombinant phage to produce stable lysogens and induce endogenous production of a foreign quorum-sensing signal.

### Production of c2HDA by the recombinant lysogenic phage reduces the expression of *Salmonella* invasion genes

**Fig 2:**
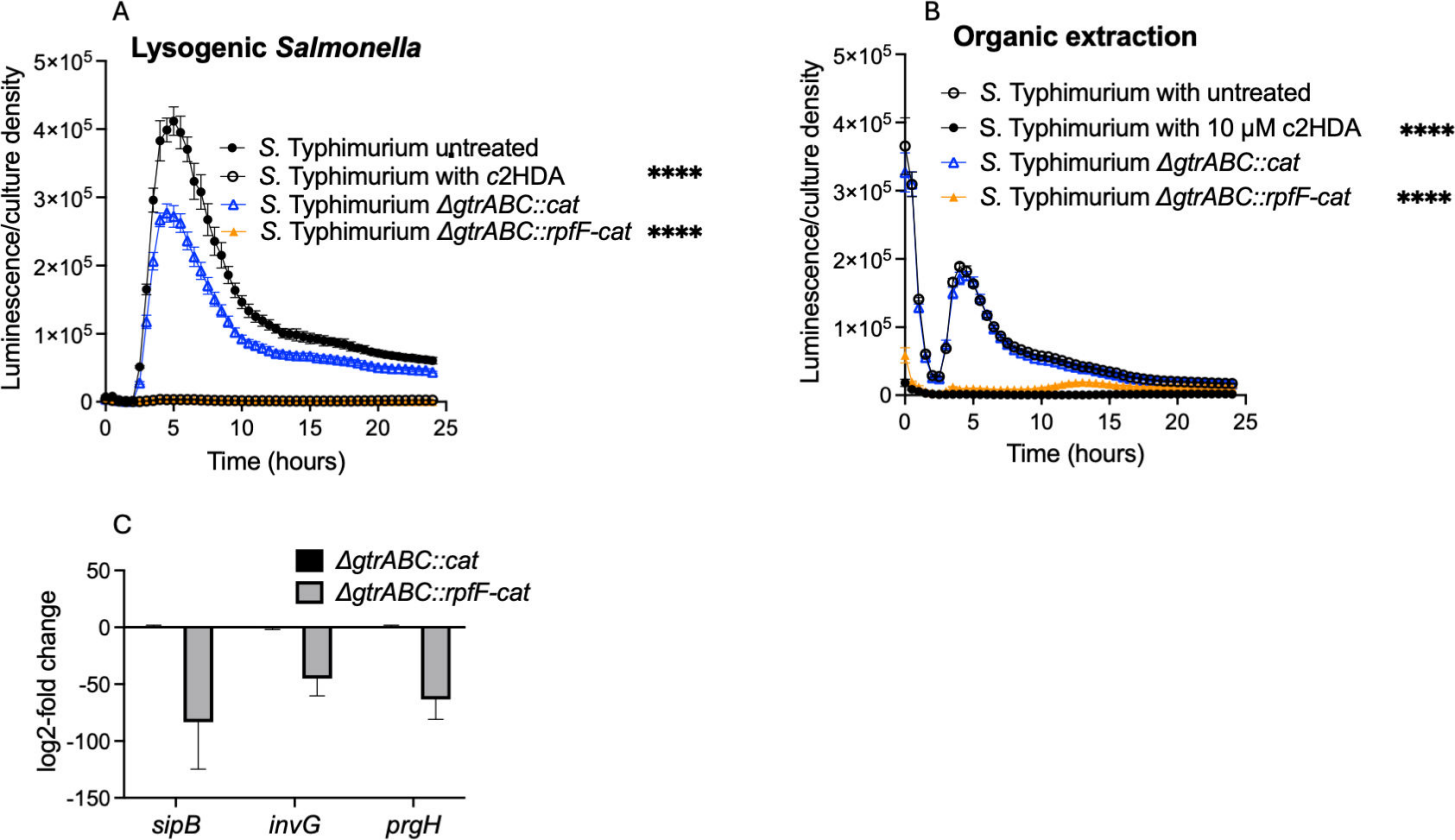
P22 Δ*gtrABC*::*rpfF* lysogenic phage-produced c2HDA reduces expression of invasion genes. (**A**) Lysogenized phage carrying *rpfF* represses *hilA* expression in *Salmonella* grown in laboratory media. Lysogens additionally carrying P*_hilA_-luxCDABE* were cultured under SPI1-inducing conditions. Data are presented as mean luminescence corrected for culture density for five biological replicates. (**B**) Organic extracts from lysogenized phage carrying *rpfF* repress *hilA* expression. Culture supernatants were organically extracted, dissolved in ethanol, and added to growing cultures of the WT strain carrying a P*_hilA_-luxCDABE* reporter fusion. (**C**) Lysogenized phage reduces message levels of invasion genes. Strains carrying phage with and without *rpfF* were cultured under SPI1 inducing conditions; mRNA was extracted and quantified by qRT-PCR. Results were normalized using the *rpoD* as the housekeeping gene. Specific primers used are listed in Table 1. Gene expression is presented as log-2-fold change. Error bars represent standard deviations of three biological replicates. Asterisks denote significant differences from the control (****P < 0.0001). Statistical significance was tested using Student’s *t*-test, and the Mann-Whitney test was applied to compare ranks.

When supplied exogenously, members of the DSF class of quorum-sensing molecules, including c2HDA itself, are known to inhibit *Salmonella* invasion of epithelial cells, a function required for virulence (9). We sought to determine whether this signal would act similarly when produced endogenously by *Salmonella* itself. To test this, we first measured the expression of *hilA*, which encodes an activator of the invasion cascade (27), using a *hilA-luxCDABE* transcriptional reporter fusion. Wild-type *Salmonella*, without lysogenized P22, demonstrated the characteristic expression of *hilA* in laboratory medium, with a rapid induction and peak expression during exponential growth (9) (Fig. 2A). A strain carrying the control phage, with a *gtrABC* deletion and associated chloramphenicol-resistance gene but lacking *rpfF* (Δ*gtrABC*::*cat*), showed a modest but significant reduction in peak *hilA* expression of 30%. In the c2HDA-producing lysogen (Δ*gtrABC::rpfF*-*cat*), however, *hilA* expression was reduced profoundly, by 132-fold at peak expression, in comparison to the control lysogen strain. This repression was similar in its proportion to that caused by the addition of 20 μM c2HDA to the medium (115-fold). The presence of neither lysogenic phage, however, affected the growth of the strains, demonstrating that the effects on *hilA* expression were independent of growth-phase induction. To ensure that the effects observed were, in fact, due to the production of a diffusible signal of the DSF class, we performed an organic extraction on the culture supernatant. When added to growing cultures of the *hilA-luxCDABE* reporter strain, extract obtained from the Δ*gtrABC::rpfF*-*cat* lysogen strain reduced peak *hilA* expression by 19-fold, compared to extract from the Δ*gtrABC*::*cat* lysogen control strain, which did not differ from the untreated control (Fig. 2B). By comparison, the addition of 10 μM c2HDA reduced expression by 156-fold in this assay.

**TABLE 1:** Primers

Primer name	Sequence
RT-PCR-*sipB*-F	ACCTGATTCTGAGAGGCGGC
RT-PCR-*sipB*-R	AGCGACAGAGGCGAAAGAGG
RT-PCR-*prgH*-F	TGCTGCGGTAACATCGTCCA
RT-PCR-*prgH*-R	GCCGCGTAAACCCGTTTTCT
RT-PCR-*invG*-F	GGGTTGCTCTTCTCCCTGCA
RT-PCR-*invG*-R	TGAGCTGGGACGCCAGAAAA
*rpfF-gtrA*-F1	CAATTTGTAGTGCTACACTTCAGACCTTTCCGAATCCGCTGATTTTCATAAACGCACAGCAAACACCACGTCGACCCTATCA
*rpfF-gtrA*-F2	GATCGGTAACAACGATCAATTAACATGCATTATATAGATAAAAACAACGCACAGCAAACACCACGTCGACCCTATCA
*rpfF-gtrC*-R1	GGTGTAAACACCCATTTTTATTTTATGTTAAATATTCTATAGCGATTGTGTAGGCTGGAGCTGCTTC
*rpfF-gtrC*-R2	CTAATTAAACCTAACAACTATGGTTTCCCCTACAACACCAATATCGATTGTGTAGGCTGGAGCTGCTTC
*rpfF* x P-*gtrA*-F	CAATTTGTAGTGCTACACTTCAGACCTTTCCGAATCCGCTGATTTTCATAATGTCTGTTTTCAACCAGTCTAC
*gtrABC*-*cat*-F	TGCTACACTTCAGATCTTTCCGAATCCGCTGATTTTCATACCATGGTCCATATGAATATCCTCCTTAGTTC
*gtrABC*-*cat*-R	TGTAAACACCCATTTTTATTTTATGTTAAATATTCTATAGGATTGTGTAGGCTGGAGCTGCTTC
*gtrABC*-chk-R	TATCCGCGCTAATAGTTTCGGCTA

To determine whether repression of the activator *hilA* elicited effects on the invasion genes it controls, we compared their expression in the Δ*gtrABC::cat* and Δ*gtrABC::rpfF-cat* lysogen strains using qRT-PCR. Both *prgH* and *invG* encode components of the *Salmonella* SPI1 needle complex (28), required for secretion of effector proteins into epithelial cells, while *sipB* encodes one of those secreted effectors (29). Expression of all these genes was greatly reduced in the Δ*gtrABC::rpfF-cat* lysogen strain, 69-, 42-, and 60-fold (for *sipB*, *invG,* and *prgH*, respectively), when compared to the isogenic lysogen harboring Δ*gtrABC::cat* that does not produce c2HDA, demonstrating that production of c2HDA through infection by a recombinant phage can efficiently repress functions required for *Salmonella* virulence (Fig. 2C).

### Endogenously produced c2HDA can act as an exogenous signal

**Fig 3:**
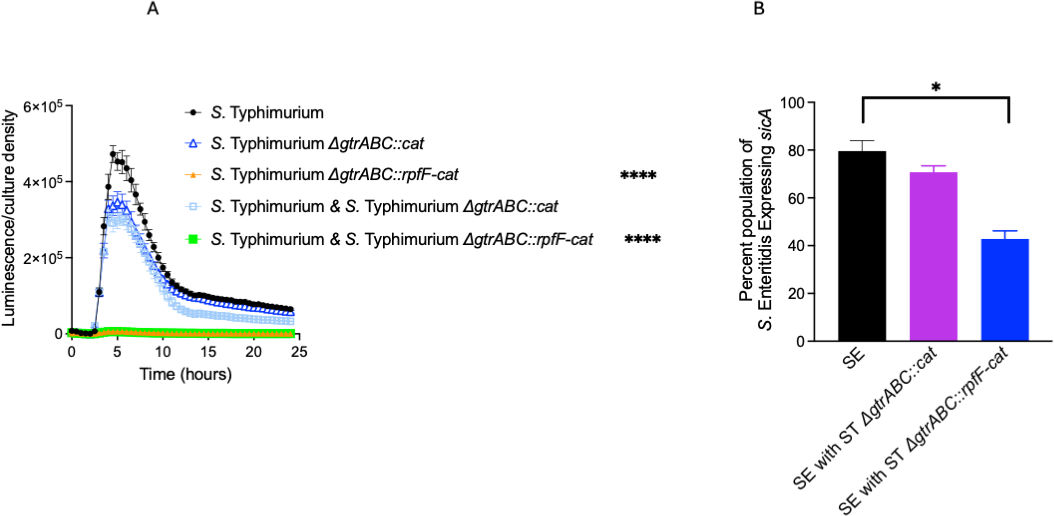
Strains carrying lysogenized phage with *rpfF* produce c2HDA that exogenously represses invasion genes in neighboring bacteria. (**A**) Co-culture assay of wild type carrying P*_hilA_-luxCDABE* reporter fusion and strains harboring lysogenized phage with or without *rpfF*. As controls, the wild-type and the lysogenized phage strains both carrying the P*_hilA_-luxCDABE* were cultured separately for comparison. Error bars represent standard deviations of five biological replicates. (**B**) Lysogenized phage produce c2HDA that can exogenously repress invasion-gene expression in *S*. Enteritidis. *S*. Enteritidis strain carrying a *sicA*-GFP reporter fusion and constitutively expressing BFP was co-cultured with an *S*. Typhimurium carrying lysogenic phage with or without *rpfF*. The number of bacteria expressing GFP was assessed by flow cytometry, and BFP was used to gate for *S*. Enteritidis. Abbreviations: ST, *Salmonella* Typhimurium and SE*, Salmonella* Enteritidis. Data are presented as percentage of bacteria expressing *sicA* for five biological replicates. Asterisks denote significant differences from the control (*P < 0.05, ****P < 0.0001). Statistical significance was tested using the Student’s *t*-test, and the Mann-Whitney test was applied to compare ranks.

Although these results demonstrate that c2HDA is produced within the cytoplasm of lysogenized strains, it is possible that *Salmonella* also secretes this signal into its surrounding environment to affect the expression of neighboring bacteria, as we have previously found for recombinant *rpfF*-expressing *E. coli* (19). To test this, we used a co-culture approach. The reporter strain in this case carried the *hilA-luxCDABE* transcriptional reporter fusion in an otherwise wild-type strain background and was grown together and in equal proportion with strains harboring the c2HDA-producing or control lysogenic phage. We then compared *hilA* expression to that of the two lysogenized strains that harbor their own *hilA-luxCDABE* fusions, described above. The reporter strain grown in co-culture with the control lysogen demonstrated a modest reduction in *hilA* expression, by 33%, similar to that of the strain carrying both the control phage and the reporter together. Co-culture of the c2HDA-producing lysogen, however, severely reduced the expression of *hilA* in neighboring bacteria, by 55-fold compared to the control lysogen (Fig. 3A). This reduction was, in fact, indistinguishable from that found through endogenous production of c2HDA by the strain harboring both the c2HDA-producing phage and the *hilA* reporter fusion (56-fold reduction). These results suggest that c2HDA produced by the recombinant phage is secreted into the culture medium, affecting expression of virulence genes in nearby *Salmonella*. Alternatively, however, the phage itself might infect these neighboring bacteria. Although the phage has been engineered to promote lysogeny, lytic growth can and does occur, raising the possibility that transfer of *rpfF* to naïve hosts induces endogenous c2HDA production. To test this possibility, we utilized *Salmonella* serovar Enteritidis, as phage P22 infects this serovar very poorly due to the activity of a restriction system not found in *S*. Typhimurium (30). We employed a dual-fluorescent *Salmonella* Enteritidis reporter strain constitutively producing blue fluorescent protein (BFP) used to determine total numbers of *Salmonella*, and encoding the gene for GFP linked to the promoter of the invasion gene *sicA* within the chromosome, assessing the proportion of the population that expressed *sicA* by flow cytometry (31). When grown alone in culture medium, 84% of the population of the *S*. Enteritidis reporter strain demonstrated detectable expression of *sicA* (Fig. 3B). Co-culture with the *S*. Typhimurium control lysogen strain lacking *rpfF* produced a small reduction in *sicA* expression to 73% of the population. Co-culture of *S*. Enteritidis with the *S*. Typhimurium strain bearing the c2HDA-producing prophage, however, significantly reduced the *sicA*-expressing population to 45%. These results, taken together, thus demonstrate that infection of *Salmonella* with a phage encoding *rpfF* and producing c2HDA can repress genes required for virulence both directly, through endogenous action of the quorum-sensing signal, and indirectly, by effects on the *Salmonella* bacteria that share their environment but have not themselves been infected.

### Production of c2HDA by infection with a recombinant phage inhibits *Salmonella* invasion and consequently inflammation

**Fig 4:**
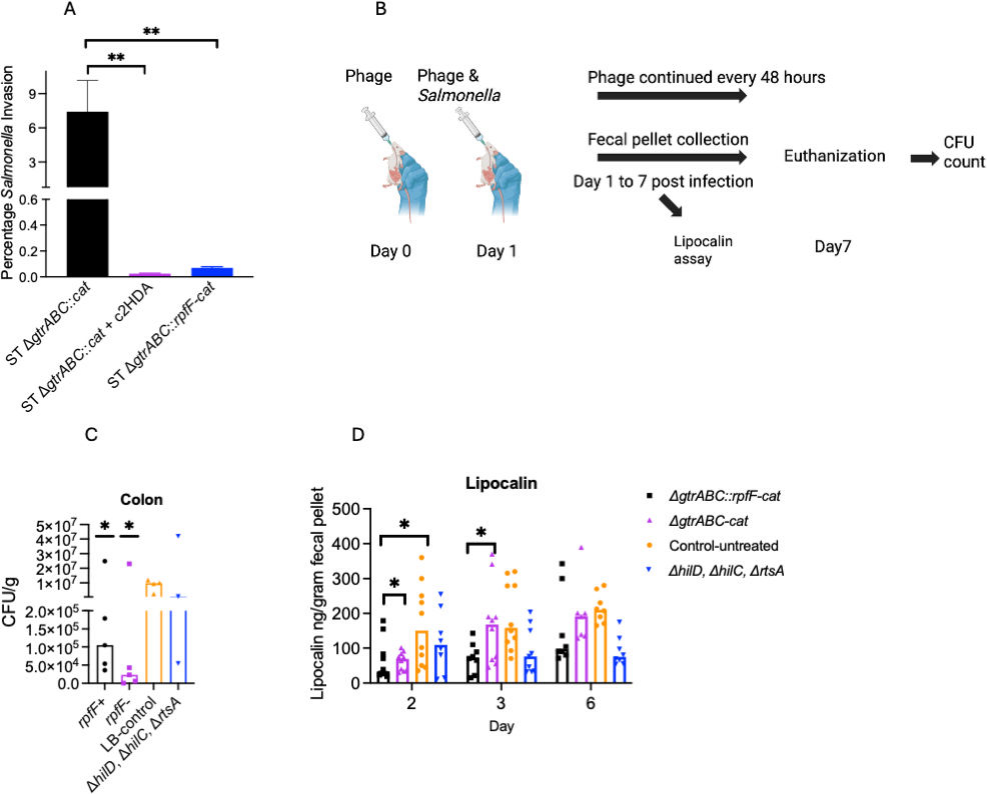
c2HDA produced by lysogenized phage inhibits invasion of epithelial cells and reduces inflammation in the gut. (**A**) Invasion of HEp-2 cells by *Salmonella* carrying lysogenized phage with or without *rpfF*. Data are presented as the percentage of *Salmonella* invading HEp-2 cells. Error bars denote standard deviation of five biological replicates. Asterisks denote significant differences from the untreated control (**P < 0.01, *P < 0.05). Abbreviations: ST*, Salmonella* Typhimurium. (**B**) Lysogenized phage carrying *rpfF* reduces inflammation in the gut. Schematic showing the procedure for treating mice with the recombinant phage. (**C**) *Salmonella* load in the colon at day 7 post-infection. Asterisks denote significant differences from the mice inoculated with the wild type but not treated with phage. (**D**) Inflammation levels in the fecal pellets. Inflammation was assessed using the lipocalin-2 assay. Data are presented as lipocalin per gram of fecal pellet. Each group contained nine mice, and asterisks denote significant differences between indicated groups (*P < 0.05). Statistical significance was tested using the Student’s *t*-test, and the Mann-Whitney test was used to compare ranks.

Repression of *Salmonella* invasion genes can reduce the ability of this pathogen to penetrate the intestinal epithelium, an early required function for both *Salmonella* carriage and disease. To test whether c2HDA production by a lysogenized phage reduced invasion, we used a well-established gentamicin-protection cell culture assay. HEp-2 cells were inoculated with *Salmonella* strains, treated with gentamicin to eliminate extracellular bacteria, and then lysed to assay the number of bacteria that had invaded the cells. We found that for the control strain carrying the Δ*gtrABC::cat* lysogenized phage, 7.4% of the inoculated population invaded cells (Fig. 4A). For the c2HDA-producing Δ*gtrABC::rpfF-cat* lysogen, however, invasion was reduced by more than 100-fold, to 0.07%. As a control, we grew the Δ*gtrABC::cat* control strain in the presence of 20 μM c2HDA prior to the assay, which reduced invasion by >300-fold, to 0.02%. The production of c2HDA and its repression of virulence genes thus has the functional consequence of reduced cell invasion.

Our data demonstrated that a lysogenized phage bearing *rpfF* can reduce penetration of *Salmonella* into epithelial cells through its repressive effects on invasion genes. We therefore anticipated that lysogenized phage would reduce expression of *Salmonella* virulence genes in the gut and consequently its ability to cause inflammation and colonize the gut. To test the effects of the phage under native gut conditions without alterating gut hemeostasis, we used 129X1/SvJ mice that can be colonized by *Salmonella* without pre-treatment with antibiotics (32). We administered the Δ*gtrABC::rpfF-cat* phage or the Δ*gtrABC-cat* phage to mice and then infected them with *Salmonella* (Fig. 4B). Phage treatment was continued every 48 h for 7 days. We found that administration of either the phage harboring *rpfF* or the control phage without it greatly and similarly reduced *Salmonella* colonization compared to untreated controls (Fig. 4C), by ~93- and 420-fold. This may be attributed to the ability of the P22 phage to lyse *Salmonella*. While we engineered the recombinant phage for lysogeny, it is possible that conditions in the gut could induce lytic growth, resulting in reduction of *Salmonella* viability. Inflammation levels were then tested using fecal pellets for levels of lipocalin-2, a common marker of inflammation induced by *Salmonella*. We found that the phage carrying *rpfF* reduced inflammation levels by ~5-fold, significantly more than by the isogenic phage bearing no *rpfF*, which reduced lipocalin-2 by ~3-fold (Fig. 4D). Invasion-gene expression is controlled by the central AraC-type transcriptional regulators HilD, HilC, and RtsA (17). Mutants of these regulators have reduced expression of effectors that are required for *Salmonella* to elicit inflammation in the gut (33, 34). As HilD, HilC, and RtsA are targets of c2HDA (9, 15, 35), we compared the levels of inflammation to those of mice infected with a Δ*hilD*, Δ*hilC*, Δ*rtsA* mutant strain. We found that inflammation in mice treated with phage carrying *rpfF* was reduced to a level close to that of mice that received the Δ*hilD*, Δ*hilC*, Δ*rtsA* strain (Fig. 4D). This indicates that orally administered recombinant phage bearing *rpfF* can infect *Salmonella* in the gut and induce it to produce c2HDA, repressing virulence effectors and thus reducing inflammation.

## DISCUSSION

Here, we have described a bacteriophage engineered to deliver to *Salmonella* a single gene that induces this pathogen to repress its own virulence. Rather than attempting to kill the organism, this form of phage therapy instead seeks to prevent *Salmonella* survival and proliferation by eliminating a function required for colonization of an animal host. This is achieved through the endogenous production of a quorum-sensing molecule, c2HDA, normally used by *Salmonella* as an exogenous signal to modulate its virulence.

c2HDA can be found in the animal intestine and therefore likely represents a means of interspecies chemical signaling, through which *Salmonella* “eavesdrops” on the quorum-sensing of neighboring bacteria to recognize its environment and modulate its gene expression, maximizing its own survival. The potential to use this system to mitigate salmonellosis therefore requires that c2HDA be delivered to *Salmonella* within the animal intestine in concentrations greater than those that occur naturally, essentially undermining the carefully controlled virulence program of this pathogen. If provided directly through oral inoculation, members of the DSF class of quorum-sensing molecules, being long-chain fatty acids, would be rapidly absorbed by the enterocytes of the small intestine. To circumvent this problem, we have previously created a recombinant probiotic *E. coli* strain that can readily colonize the gut and deliver c2HDA to the surrounding environment (19). The production of this molecule within *Salmonella* itself holds promise to improve the efficiency of this mitigation strategy, due to the high local concentrations likely to ensue.

The development of resistance to bacteriophage by their bacterial targets remains a persistent obstacle to the use of phage therapy as a tool for disease treatment or prevention. *Salmonella* has indeed been shown to develop resistance to P22, the phage used here, by multiple means (36). The proliferation of such mutants might, however, be mitigated at least in part by the loss of fitness or virulence that such mutations can impart (37). Additionally, P22 is but one of many related phage, sensitivity to which has long been used to determine the serotypes of *Salmonella* isolates (38). The availability of a well-characterized set of such phage in a genetically tractable organism such as *Salmonella* might provide useful alternatives to P22 that could extend and augment this approach.

Treating or preventing infection by enteric pathogens is particularly difficult due to the complex bacterial milieu that surrounds them. The use of broad therapies such as antibiotics risks wholesale disruption of the intestinal microbiota, resulting in widespread dysbiosis with severe consequences to health. The use of phage instead offers the possibility of a highly targeted disease-mitigation strategy. Infection by P22 and other phage of *Salmonella* can be directed to specific serovars and, in some cases, to strains within serovars. They therefore offer the possibility of directed prevention, targeting the *Salmonella* types found in specific animal species, locations, and production systems.

## MATERIALS AND METHODS

### Strains, plasmids, and growth conditions

*Salmonella enterica* subsp. *enterica* serovar Typhimurium 14028s and *Salmonella enterica* subsp. *enterica* serovar Enteritidis MD15, and mutants thereof, were used throughout, as listed in Table 2. All bacterial cultures were grown in LB broth at 37°C with aeration unless otherwise described. The derivative of phage P22 used in these studies was created by selecting transposon mutants that exhibited lysogeny with high frequency. A *S*. Typhimurium strain was constructed harboring two plasmids: pNK2887 has a defective *Tn*10 derivative carrying kanamycin resistance and an outward-facing IPTG-inducible promoter (26). Its transposase is encoded on the plasmid, but not within the transposon, and is also inducible with IPTG. Thus, transposition can be induced, but once it occurs, it results in a stable transposon. pMS421 encodes *lacI^q^* for overproduction of *lac* repressor (39). This strain was grown with 1 mM IPTG to induce transposition, wild-type P22 was added, and the strain was grown with aeration overnight. A phage lysate was prepared by the addition of chloroform and was used to infect wild-type *S*. Typhimurium. Strains harboring lysogens were selected by growth on LB agar with 50 μg/mL kanamycin. Phage with efficient lysogeny was identified by introducing lysogenic phage into strains with null mutations of *katE* and *rpoS* and inducing lytic growth with hydrogen peroxide (2.5 mM) overnight, harvesting the resulting phage lysate with chloroform, and transducing wild-type *S*. Typhimurium, comparing kanamycin-resistant colonies to plaque-forming units. *rpfF* was integrated into the phage genome under the control of the P2 promoter, reported to provide moderate expression (Addgene) (40), along with an associated chloramphenicol-resistance gene. These genes were introduced into the *gtrABC* operon of phage P22 using the one-step inactivation method as previously described (41).

**TABLE 2:** Strains and plasmids

Strain/plasmid	Genotype/sequence	Reference
CA32	*Salmonella enterica* serovar Typhimurium 14028s	ATCC
CA5395	P22 *sieB-esc-Tn10-kan* lysogen	This study
CA2286	*S*. Typhimurium, P*_hilA_-luxCDABE-tet*	(7)
CA5594	Δ*gtrABC-cat*	This study
CA5560	Δ*gtrABC::rpfF-cat*	This study
CA5606	Δ*gtrABC*	This study
CA5602	Δ*gtrABC::rpfF*	This study
CA5598	Δ*gtrABC-cat,* P*_hilA_-luxCDABE-tet*	This study
CA5567	Δ*gtrABC::rpfF-cat,* P*_hilA_-luxCDABE-tet*	This study
CA5484	*S*. Enteritidis *sicA→GFP phoN→BFP malxY::kan*	This study
CA5745	Δ*gtrABC,* Δ*katE,* Δ*rpoS::cat*	This study
CA5744	Δ*gtrABC::rpfF,* Δ*katE,* Δ*rpoS*	This study
CA4434	*S*. Typhimurium, Δ*malXY::cat*	(34)
CA3996	*S*. Typhimurium, Δ*hilD*, Δ*hilC::kan*, Δ*rtsA::cat*	(2)

### Fatty acid extraction

Strains were cultured in LB broth overnight, and fatty acids produced by the bacterial strains were extracted using chloroform. Bacterial supernatants were mixed with two volumes of chloroform and mixed by rotation for 20 min. The chloroform phase was carefully decanted into a glass container. Extraction was repeated twice and the extracts pooled. Chloroform was evaporated, and the fatty acids extracted were air-dried. Extracts were derivatized and analyzed by GC-MS or dissolved in ethanol for treating bacterial cultures.

### Identification of signal

For signal identification, extracted fatty acids were derivatized overnight with a 1:1 mixture of hexane and 3-(trifluoromethyl) phenyltrimethylammonium hydroxide (TCI chemicals). A volume of 0.05 mL extract in ethanol was derivatized in a volume of 0.2 mL of the derivatizing agent. Derivatized samples were separated on an Agilent 19,091B-102 Ultra 2 35 m × 200 µm × 0.33 µm column mounted on a Thermo GC-Orbitrap system coupled to a mass spectrometer. Commercially acquired c2HDA was used as a standard for retention time analysis and mass fragmentation.

### Luciferase assays

Strains carrying *luxCDABE* reporter fusions on a plasmid were grown overnight in LB with the necessary antibiotics. Overnight cultures were diluted 100-fold into M9 minimal medium with glucose, antibiotics, and 1 mM nonanoic acid (added to repress SPI invasion gene expression to eliminate background luminescence) and grown overnight. Bacteria were inoculated at a starting optical density at 600 nm (OD_600_) of 0.02 into 150 µL of LB containing 100 mM morpholinepropanesulfonic acid (pH 6.7). Luminescence was measured in a BioTek Synergy H1 microplate reader at 37°C for 24 h.

### qRT-PCR

Lysogens were cultured in LB broth as above for 4 h. RNA was extracted using the TRIzol reagent according to the manufacturer’s instructions. Briefly, 0.25 mL of bacterial sample was mixed with 1 mL of TRIzol. The sample was then lysed by bead-beating in 2 mL glass bead-preloaded microtubes (Omni International). Samples were centrifuged to separate the beads. The supernatant was mixed with 0.2 mL of chloroform, incubated for 3 min, and then centrifuged. The RNA sample was precipitated using isopropanol and washed twice with 75% ethanol. The dried sample was reconstituted in RNase-free water. qPCR was performed using the iTaq Universal SYBR Green One-step kit (Bio-Rad) according to the manufacturer’s instructions. For each sample, 100 ng of RNA was used, and experiments were performed with three technical replicates. Reverse transcription was performed at 50°C for 10 min, and the cDNA was initially denatured for 5 minutes at 95°C. Quantitative PCR was performed using specific primers for the genes to be tested. Results were normalized by comparing them with the housekeeping gene *rpoD*. All tests were performed in triplicate.

### Flow cytometry

Strains carrying *sicA*-GFP and additionally constitutively expressing BFP were cultured overnight in LB broth. Bacterial cultures were pelleted and resuspended in 4% paraformaldehyde in PBS and fixed for 30 min at 4°C. Flow cytometry was performed as previously described (31). Recovered bacteria were analyzed for blue fluorescent (BFP) and green fluorescence (GFP) using an Attune analyzer NxT flow cytometer (Thermo Fisher). *Salmonella* was identified by BFP expression, and GFP was used to monitor invasion-gene expression.

### Invasion assay

Invasion was determined using the gentamicin-protection assay as previously described (9). Strains to be tested were cultured overnight in LB. Bacteria were washed with PBS, and ~2 × 10^6^ were added to HEp-2 cells to maintain a multiplicity of infection of 10. Plates were centrifuged for 10 min at 100 × *g* and incubated for 1 hour at 37°C. Plates were then washed, and gentamicin was added at a concentration of 20 μg/mL. After 1 h of incubation, cells were washed and lysed with 1% Triton X-100. Lysates were plated for enumeration of recovered intracellular bacteria. Percentage invasion of the strains was calculated by comparing the number of bacteria inoculated with that of bacteria recovered.

### Animal experiments

The 129X1/SvJ mouse model was used for testing the activity of lysogenized phage in the gut. This is a well-established mouse model that is susceptible to *Salmonella* colonization without the use of antibiotics and has been used for long-term infections (32). Mice 6–8 weeks old were acquired from the Jackson Laboratories. A total of 1 × 10^8^ plaque-forming units of extracted phage were administered in 100 µL by oral gavage a day before *Salmonella* infection and every other day after infection. Mice were inoculated with a total of 1 × 10^8^ CFU of *Salmonella* strains in 100 µL PBS. Fecal pellets were collected for plating to assess colonization, and inflammation was tested by lipocalin-2 assay (R&D systems) according to manufacturer’s guidelines.

### Statistical analysis

Results are presented as means with standard deviations. Comparisons between controls and tests were evaluated with GraphPad Prism 10 using the Student’s *t*-test.
